# Relationship Between Estradiol Levels Measured on the Initiation Day of GnRH Antagonist Treatment and Pregnancy Outcomes in Patients Receiving the Antagonist Protocol

**DOI:** 10.3390/medicina61040741

**Published:** 2025-04-17

**Authors:** Pınar Karaçin, Runa Özelçi, Enes Kumcu, Dilek Kaya Kaplanoğlu, Serdar Dilbaz, Yaprak Üstün

**Affiliations:** 1 Department of Gynecology, Etlik City Hospital, Ankara 06010, Türkiye; enes_kumcu@hotmail.com; 2 Department of Gynecology, Etlik Zubeyde Hanim Training and Research Hospital, Ankara 06010, Türkiye; runakara@gmail.com (R.Ö.); sdilbaz@gmail.com (S.D.);; 3 Department of Gynecology, Adana Yüreğir State Hospital, Adana 01240, Türkiye; dilekkaplanoglu@gmail.com

**Keywords:** in vitro fertilization, embryo transfer, gnrh, antagonist protocol, serum estradiol, pregnancy

## Abstract

*Background and Objectives:* The aim of this study is to evaluate the relationship between serum estradiol (E2) levels measured on the day of antagonist administration and live birth rates (LBRs) in women undergoing IVF-ET with an antagonist protocol. *Materials and Methods:* Data from women who underwent IVF-ET with an antagonist protocol between 2011 and 2023 were retrospectively analyzed. Patients were divided into five groups on the basis of serum E2 levels measured on the day of antagonist administration (Group I: E2 < 400 pg/mL, Group II: 400 ≤ E2 < 650 pg/mL, Group III: 650 ≤ E2 < 800 pg/mL, Group IV:800 ≤ E2 < 1000 pg/mL, and Group V: E2 ≥ 1000 pg/mL). The independent effect of serum E2 levels on live birth was analyzed via an adjusted regression model. *Results:* A total of 1613 patients were included in the study. The overall LBR was 32.1%. The LBRs for Groups I, II, III, IV, and V were 28.9%, 37.8%, 26.4%, 32.2%, and 34.1%, respectively (*p* = 0.017). In the adjusted regression model, serum E2 levels measured on the day of antagonist administration < 400 pg/mL (OR: 0.752, 95% CI: 0.580–0.999, *p* = 0.048) and 650 ≤ E2 < 800 pg/mL (OR: 0.595, 95% CI: 0.388–0.911, *p* = 0.011) were identified as factors that reduce the likelihood of a live birth, adjusting for age, infertility duration, body mass index (BMI), cycle number, quality of embryo, and number of embryos transferred. *Conclusions:* The serum E2 level associated with the highest LBR in women undergoing IVF-ET with an antagonist protocol was found to be in the range of 400–650. Serum E2 levels < 400 pg/mL or in the range of 650–800 pg/mL were statistically significantly associated with a reduced LBR.

## 1. Introduction

In recent years, the landscape of assisted reproductive technology (ART) has evolved significantly, with a notable surge in the utilization of gonadotropin-releasing hormone (GnRH) antagonist protocols for in vitro fertilization (IVF) [1]. This surge can be attributed to the heightened efficacy of GnRH antagonist protocols in preventing premature ovulation, offering a more controlled approach to follicular development [2,3]. Infertility, which affects a substantial proportion of the global population, has prompted the continual refinement of ART to address the diverse challenges faced by individuals aspiring to build their families. GnRH antagonist protocols have emerged as a cornerstone in this pursuit, allowing for precise control over the timing of ovulation and optimizing the chances of successful fertilization [1,3].

In applying the GnRH antagonist protocol, there are two different methods, fixed and flexible, depending on the starting time of the GnRH antagonist [4]. Although there is no significant difference in pregnancy outcomes between the two methods, the flexible method is preferred due to reduced GnRH antagonist usage [4,5]. Despite being used for years, there is no certainty about which criterion will be used to start GnRH antagonists using the flexible method. The most important criterion for starting a GnRH antagonist is follicle size, but there is no recommendation regarding serum hormone values [4,5]. The initiation of the GnRH antagonist heralds a pivotal juncture in the IVF cycle, where the suppression of endogenous gonadotropins assumes paramount importance for orchestrating a controlled and optimal stimulation of follicular development [1].

Despite the widespread adoption of GnRH antagonist protocols, there is a critical need to identify specific biomarkers that can reliably predict the success of fertility treatments [6]. Conflicting results exist regarding the effect of serum estradiol levels on pregnancy outcomes in IVF patients. Two studies have found a non-linear relationship between serum estradiol (E2) levels and clinical pregnancy [4,5]. On the other hand, several other studies have shown that serum E2 levels do not affect pregnancy outcomes [7,8,9]. The implications of various aspects of IVF outcomes have been explored, yet the intricate relationship between serum estradiol levels on the day of GnRH antagonist initiation and the probability of achieving clinical pregnancy remains an active area of investigation [4,5]. Previous studies have suggested the potential impact of estradiol on pregnancy outcomes, but a thorough examination of this relationship is essential [4].

We aimed to evaluate the relationship between serum E2 levels measured on the day of GnRH antagonist administration and live birth rates (LBRs).

## 2. Materials and Methods

This retrospective study was carried out at the Health Sciences University, Etlik Zübeyde Hanim Training and Research Hospital IVF Clinic between January 2011 and 2023 years. A total of 1625 women between 23 and 40 years old who underwent IVF cycles with antagonist protocols were included in the study. Those with a diminished ovarian reserve, polycystic ovary syndrome (PCOS), cycle cancellations for any reason, and those with unknown serum E2 levels were excluded from the study. We excluded women with PCOS and diminished ovarian reserve to ensure that extreme estradiol values would not affect the results. This study was approved by the Etlik Zübeyde Hanım Training and Research Hospital Ethical Committee (date: 31 January 2024, number: 01/12).

The data were retrospectively evaluated from hospital archive files and electronic records. Patient information, including age, obstetric history (gravidity, parity, live births, miscarriages, history of dilation/curettage, history of uterine surgery, and cesarean and vaginal delivery numbers), infertility causes, previous IVF history, serum E2 levels measured on the day of GnRH antagonist administration, dominant follicle measurement levels, collected oocyte numbers, mature oocyte numbers, fertilized oocyte numbers, embryo transfer numbers, clinical pregnancy, and LBR information were recorded. While collecting the study data, we acted in accordance with the personal data protection law applied in our country.

Body mass index (BMI), calculated as weight in kilograms divided by the square of height in meters and waist circumference (measured in centimeters on bare skin), was recorded.

The GnRH antagonist cetrorelix 0.25 mg (Cetrotide; Merck-Serono, Darmstadt, Germany) was started per day when the leading follicle diameter was 13–14 mm, and then it was continued until the administration of the ovulation trigger. When at least 3 follicles in the size of 17–18 mm were observed, the final ovulation triggering was performed by injection of recombinant human chorionic gonadotropin (r-hCG) (Ovitrelle; Merck-Serono, Darmstadt, Germany), and then 35.5–36 h later, the ovum pick-up (OPU) was planned. Patients underwent fresh embryo transfer. In fresh cycles, embryo transfer was performed 3 or 5 days after oocyte retrieval under transabdominal ultrasonographic guidance. Embryo quality classification was made according to the 2011 Istanbul ESHRE consensus [10]. Luteal phase support consisted of intramuscular progesterone at 50 mg/day and/or oral dydrogesterone at 30 mg daily from the day of oocyte retrieval until the β-hCG test. Serum β-hCG levels were measured 14 days post-oocyte pick-up, and in cases of pregnancy, luteal phase support was continued up to 10–12 weeks of gestation. Clinical pregnancy (CP) was defined as the presence of an intrauterine sac with fetal heart activity on transvaginal ultrasonography at 7 weeks of gestation.

Serum levels of follicle stimulating hormone (FSH), luteinizing hormone (LH), E2, and progesterone were measured via the chemiluminescence method with a Cobas E-800 (Roche, Basel, Switzerland) hormone autoanalyzer. Patients were arbitrarily categorized into five groups on the basis of their serum E2 levels measured on the day of GnRH antagonist administration (Group I: E2 < 400 pg/mL, Group II: 400 ≤ E2 < 650 pg/mL, Group III: 650 ≤ E2 < 800 pg/mL, Group IV: 800 ≤ E2 < 1000 pg/mL, and Group V: E2 ≥ 1000 pg/mL). The primary outcome of this study was to measure the LBR according to serum E2 levels measured on the day of GnRH antagonist administration.

During the preparation of this manuscript, in order to improve the quality of the English writing of the article, support was received from the Jenni AI program, Altum Inc., Wilmington, DE, USA. The scientific data are original. The accuracy of the support received from AI has been checked by us. The authors have reviewed and edited the output and take full responsibility for the content of this publication

### Statistical Analysis

The descriptive statistics of continuous variables were presented as mean ± standard deviation, and categorical variables were expressed as frequencies (%). An ANOVA test was employed for the comparison of continuous variables between groups, while a chi-square or Fisher’s Exact test was utilized for the comparison of categorical variables. Univariate regression analysis was conducted to identify factors that could affect live birth. Following the identification of statistically significant variables from the univariate analysis, a multivariate logistic regression model was constructed, incorporating serum E2 levels measured on the day of GnRH antagonist administration, to assess whether serum E2 level is an independent predictive factor for live birth. All tests were two-tailed, and *p* < 0.05 was considered statistically significant. Statistical analyses were conducted using IBM Corp. Released 2017. IBM SPSS Statistics for Windows, Version 25.0. Armonk, NY, USA: IBM Corp.

## 3. Results

Patients were categorized into five groups based on serum E2 levels measured on the day of GnRH antagonist administration (Group I: E2 < 400 pg/mL, *n*: 589, Group II: 400 ≤ E2 < 650 pg/mL, *n*: 399, Group III: 650 ≤ E2 < 800 pg/mL, *n*: 174, Group IV: 800 ≤ E2 < 1000 pg/mL, *n*: 155, Group V: E2 ≥ 1000 pg/mL, *n*: 296). A comparison of the clinical characteristics among the groups is presented in Table 1. The mean ages of the groups were similar (*p* = 0.210). The average BMI of Group IV was lower than that of Group I (*p* = 0.001). The mean antral follicle count was highest in Group V but lowest in Group I. The average dose of gonadotropin administered was highest in Group I and lowest in Group V. The mean numbers of retrieved, mature, and fertilized oocytes were highest in Group V and lowest in Group I. The overall clinical pregnancy rate was 36.1%. The clinical pregnancy rates for Groups I, II, III, IV, and V were 32.9%, 41.1%, 31.0%, 36.1%, and 38.5%, respectively (*p* = 0.048). The highest clinical pregnancy rate was observed in Group II (41.1%). The LBR for Groups I, II, III, IV, and V were 28.9%, 37.8%, 26.4%, 32.2%, and 34.1%, respectively (*p* = 0.017). The highest clinical pregnancy rate was observed in Group II (37.8%).

### Factors Influencing Clinical Pregnancy

Univariate logistic regression analysis revealed that the LBRs decreased with age, but they increased with increasing antral follicle count. Furthermore, statistically significant differences in the LBRs were observed among the groups on the basis of the serum E2 levels measured on the day of GnRH antagonist administration (Table 2).

The multivariate logistic regression analysis revealed that, when considering the Group II E2 range as a reference, the likelihood of LBR decreased in Group I and Group III (Table 3).

## 4. Discussion

The findings of our study contribute to the growing body of knowledge in the field of ART, particularly in the context of GnRH antagonist protocols for IVF. The increased utilization of GnRH antagonist protocols in recent years is reflective of the continuous efforts to refine and optimize fertility treatments, aiming for improved success rates in achieving clinical pregnancies and live births [11].

Our investigation into the relationship between serum E2 levels on the day of GnRH antagonist initiation and clinical pregnancy and live birth rates provides valuable insights into the literature. Serum E2, a key regulator of the female reproductive system, has long been implicated in various aspects of IVF outcomes. Our study reinforces the importance of considering serum E2 levels as a potential biomarker for predicting pregnancy outcomes. According to our multivariate logistic regression analysis, in terms of Group II E2 levels, both Group I (E2 < 400 pg/mL) and Group III (650 ≤ E2 < 800 pg/mL) patients presented decreased odds of a live birth. This underscores the importance of maintaining a delicate hormonal balance during ovarian stimulation, where deviations, either too low or too high, may impact the likelihood of a successful pregnancy. Fine-tuning stimulation protocols to keep E2 levels within an optimal range can be crucial for treatment success. The stratification of patients into distinct groups based on serum E2 levels highlighted significant differences in clinical pregnancy rates and live birth rates. This approach provides a foundation for a more personalized and targeted treatment protocol. Group II, with serum E2 levels between 400 and 650 pg/mL, exhibited the highest clinical pregnancy and live birth rate, suggesting an optimal range for successful outcomes. On the other hand, there was no difference in clinical pregnancy and live birth rates between Group II and Groups IV and V. This supported a non-linear positive relationship between serum E2 and clinical pregnancy, similar to previous studies with positive results [4,5].

In a few studies evaluating the relationship between serum E2 levels and clinical pregnancy, findings similar to ours have been obtained [4,5]. Wang et al. assessed the relationship between serum E2 levels measured on the initiation day of GnRH antagonist therapy and clinical pregnancy in 1493 IVF-ET cycles due to single tubal factor infertility [5]. They identified a non-linear relationship between serum E2 levels and clinical pregnancy. They demonstrated that serum E2 levels below 436 or above 894 reduced the likelihood of clinical pregnancy [5]. Similar relationships were also reported in a study by Schumacher et al., who reported that the highest clinical pregnancy rate was observed in patients with serum E2 levels between 500 and 599 pg/mL and that serum E2 levels exceeding 1100 pg/mL decreased the likelihood of clinical pregnancy [4].

In IVF-ET protocols, excessive ovarian stimulation can sometimes lead to elevated E2 levels, which may adversely affect embryo implantation [12]. Serum E2 levels play a critical role in rendering endometrial receptivity [13]. The optimal chances of pregnancy require the endometrium to possess specific thickness, vascularity, and receptivity. Excessively high or low E2 levels can disrupt endometrial receptivity, consequently leading to implantation failure [13]. Elevated E2 levels can negatively impact follicular function and reduce oocyte quality, resulting in decreased embryo quality and, subsequently, pregnancy failure [14,15]. On the other hand, study results also indicate that high E2 levels do not negatively affect clinical pregnancy outcomes [8,9,16]. Chen et al.’s study evaluating 697 IVF-ET cycles showed that high peak E2 levels do not negatively affect clinical pregnancy and may even have a positive effect on those who underwent ET on day 5 [8]. Karatasiou et al.’s systematic review and meta-analysis did not support the presence of an association between the probability of pregnancy after ovarian stimulation for IVF using GnRH analogs and gonadotrophins and serum Ε2 levels on the day of triggering final oocyte maturation with hCG. This finding was present in all Ε2 threshold groups evaluated [9]. However, this meta-analysis’s conclusion emphasizes that the trend of a decrease in pregnancy rates parallel with an increase in serum Ε2 levels needs to be further evaluated [9].

The observed decrease in clinical pregnancy rates and live birth rates with increasing age aligns with the well-established understanding of age-related decreases in fertility [17]. As age increases, oocyte quality and the ovarian reserve typically decrease, affecting the overall success of IVF treatments [18]. This underscores the importance of personalized treatment approaches for different age groups, with careful consideration of individualized factors that may impact treatment outcomes. On the other hand, the positive association between the antral follicle count and clinical pregnancy rate emphasizes the importance of the ovarian reserve in determining IVF success [19]. Higher antral follicle counts are generally indicative of a more robust ovarian reserve, which correlates with improved oocyte quality and quantity [19,20]. Tailoring treatment strategies based on antral follicle counts can aid in optimizing outcomes for individuals with varying ovarian reserves.

In our study, only those who underwent a fresh cycle were included. It has been hypothesized that in fresh cycles, higher estradiol levels may luteinize the endometrium earlier, leading to increased progesterone and a more difficult pregnancy [21,22]. Studies show that progesterone levels may have adverse effects on pregnancy outcomes, while others show that they have no effect [23]. Baldini et al. have shown that the number of collected and mature oocytes may be higher at high progesterone levels [24].

Although a definitive mechanism linking serum E2 levels and live birth outcomes remains elusive, several potential explanations warrant consideration. Previous research [25,26] has indicated that supraphysiologic estradiol (E2) levels, a consequence of controlled ovarian hyperstimulation, may negatively impact endometrial receptivity and subsequent clinical pregnancy rates. Furthermore, the association between elevated E2 levels and adverse obstetric outcomes, such as preeclampsia, low birth weight, and infants small for gestational age, lends support to the hypothesis that high E2 is linked to impaired endometrial decidualization and suboptimal placentation. One possible mechanism involves supraphysiologic E2 inducing endometrial edema, potentially hindering uteroplacental blood flow and villous trophoblast invasion.

Additionally, elevated E2 levels may exert a negative influence on oocyte and embryo quality. While the number of oocytes retrieved and transferable embryos may increase with rising E2 levels on the day of GnRH antagonist initiation, the proportional increase in transferable embryos appears to be less than that of retrieved oocytes. Assuming that only mature oocytes are capable of fertilization and developing into transferable embryos, this discrepancy could be attributed to suboptimal follicular synchronization and impaired oocyte maturation rates associated with high E2 levels during GnRH antagonist initiation. In vitro studies have further demonstrated that exposing human embryonic stem cells to supraphysiologic E2 levels can suppress cell proliferation [5].

While our study provides valuable insights, it is essential to acknowledge its limitations. Retrospective analyses inherently pose limitations in controlling for all potential confounding variables. Additionally, the single-center nature of this study may introduce biases, and further multicenter investigations are warranted for generalizability. Although not having evaluated a specific subgroup in terms of infertility causes the study population heterogeneity, this constitutes another limitation. This diversity may have affected the results, suggesting different optimal estrogen levels for subgroups with distinct causes. The study results were influenced by the fact that luteal support was applied in two different ways in our center, intramuscularly and orally. Another limitation of the study was the difference in retrieved oocytes between the groups and the fact that the groups had lower median oocyte numbers than the recommended median of 15 oocytes for IVF. A limitation that could affect the results was the fact that the groups differed in terms of some general characteristics (such as BMI, antral follicle count (AFC), and retrieved oocytes). In our center, the start date of an LHRH antagonist is decided according to follicle size. In different centers, this decision can also be made according to E2 and LH levels. This was considered as one of the limitations of our study. Since the study was retrospective, a control arm could not be created, and the groups could not be homogenized. However, these interactions were minimized with multivariate analyses. Since no clear cut-off was given for serum E2 classification, the study groups were determined arbitrarily according to serum E2 levels. One of the strengths of the study was that E2 levels were an independent predictor for clinical pregnancy through analyses adjusted for confounding factors, such as BMI, age, and AFC.

In our study, lower live birth rates were found in Group I and Group III compared to Group II. On the other hand, no significant difference was found between Group I and Group IV or Group V. Another study found a similar non-linear relationship [5]. On the other hand, our study and the other study were retrospective, the groups were not distributed homogeneously, and the number of patients in the groups was different, which warns us to be more careful when interpreting the results we obtained. Therefore, more reliable and definite results can be obtained with prospective randomized studies.

Accumulating evidence suggests that several vitamin dysbalances, as well as nutraceutical supplementation to counteract them, may play a significant role on women’s health, especially in ovarian stimulation to retrieve better egg quality, as well as in the antagonist protocols in the case of fertility preservation, as in the case of breast cancer prevention [27,28,29,30]. In this context, the body of evidence is increasing regarding the role of various vitamin imbalances and nutritional supplements on women’s health. It is posited that these imbalances and supplements may exert noteworthy effects within antagonist protocols, particularly in scenarios such as ovarian stimulation aimed at obtaining enhanced oocyte quality, fertility preservation endeavors, and breast cancer prevention strategies. By way of illustration, the established role of inositols in insulin signaling underscores the importance of employing myo-inositol and D-chiro-inositol isomers as insulin sensitizers in the management of polycystic ovary syndrome [29,30]. Furthermore, it is suggested that alpha-lipoic acid may present utility in the therapeutic intervention of PCOS, owing to its demonstrated effects on ameliorating insulin resistance [29]. The judicious application of such nutritional supplements holds considerable promise for the realization of personalized and efficacious treatment modalities.

## 5. Conclusions

Our study illuminates the complex interaction of age, antral follicle number, and serum E2 levels in the context of GnRH antagonist protocols for IVF. Multivariate analysis showed that serum E2 levels may affect pregnancy outcomes non-linearly. These findings contribute to ongoing efforts to increase the precision and success rates of fertility treatments. Serum E2 level measured on the day of application of the GNRH antagonist protocol may be related to live birth, and perhaps applying a serum E2 level between 400 and 650 IU may be beneficial.

## Figures and Tables

**Table 1:** Comparison of clinical pregnancy and live birth rates and other characteristics among groups formed according to serum E2 levels.

		Group I E2 < 400 pg/mL	GROUP II 400 ≤ E2 < 650 pg/mL	GROUP III 650 ≤ E2 < 800 pg/mL	GROUP IV 800 ≤ E2 < 1000 pg/mL	GROUP V E2 ≥ 1000 pg/mL	*p*-Value
N		589	399	174	155	296	
Age, year		30.5 ± 5.6	30.1 ± 4.8	30.0 ± 5.0	30.9 ± 4.6	30.8 ± 4.8	0.210
Duration of infertility, years		5.9 ± 4.3	6.1 ± 4.2	5.6 ± 3.9	6.1 ± 3.9	6.0 ± 3.7	0.708
Cycle count		1.82 ± 1.07	1.85 ± 1.22	1.66 ± 1.01	1.73 ± 0.93	1.79 ± 1.13	0.348
BMI, kg/m^2^		27.5 ± 5.06	26.2 ± 4.51	26.0 ± 4.81	25.6 ± 4.30	26.3 ± 4.85	<0.001
Basal E2		44.3 ± 31.4	45.8 ± 20.6	47.7 ± 21.2	46.0 ± 26.2	47.9 ± 18.1	0.278
Antral follicle count		13.5 ± 8.5	14.7 ± 7.8	17.4 ± 8.6	18.3 ± 8.9	19.1 ± 8.7	<0.001
Gn total dose		2471 ± 1023	2068 ± 945	1819 ± 693	1780 ± 684	1679 ± 648	<0.001
Duration of stimulation, days		10.1 ± 1.5	9.7 ± 1.5	9.8 ± 1.4	9.8 ± 1.4	9.9 ± 1.6	<0.001
GnRH ant start date		5.7 ± 0.8	5.6 ± 1.0	5.8 ± 1.1	5.8 ± 1.2	5.9 ± 1.1	0.001
E2 on date of hCG, pg/mL		1603 ± 911	2159 ± 1132	2540 ± 1331	2615 ± 1265	3413 ± 1901	<0.001
P on date of hCG, ng/mL		0.86 ± 0.48	0.93 ± 1.00	0.88 ± 0.54	0.82 ± 0.42	1.04 ± 1.07	0.009
P on date of antagonist initiation, ng/ml		0.56 ± 0.37	0.60 ± 0.41	0.57 ± 0.48	0.51 ± 0.40	0.62 ± 0.61	0.086
LH on date of antagonist initiation, IU/L		4.9 ± 2.3	5.0 ± 2.4	5.3 ± 2.2	5.3 ± 3.0	5.5 ± 3.1	0.009
Endometrial thickness		10.17 ± 1.93	9.98 ± 1.89	10.00 1.79	10.12 ± 2.18	10.21 ± 2.10	0.606
Retrieved oocyte		9.57 ± 5.71	11.50 ± 6.04	14.10 ± 7.94	13.40 ± 5.91	15.41 ± 7.51	<0.001
Mature oocyte		7.11 ± 4.44	8.55 ± 4.82	10.83 ± 6.51	10.23 ± 4.47	12.01 ± 6.21	<0.001
2 pn		3.89 ± 2.78	4.73 ± 3.08	6.03 ± 4.24	5.68 ± 3.48	6.88 ± 4.35	<0.001
Transferred embryos		1.30 ± 0.49	1.35 ± 0.57	1.33 ± 0.56	1.32 ± 0.53	1.33 ± 0.44	0.667
Quality of embryos							
	Gr1	0.58 ± 0.63	0.67 ± 0.69	0.62 ± 0.67	0.77 ± 0.70	0.65 ± 0.67	0.019
	Gr2	0.48 ± 0.57	0.46 ± 0.63	0.44 ± 0.57	0.37 ± 0.60	0.44 ± 0.59	0.336
	Gr3	0.19 ± 0.41	0.17 ± 0.43	0.25 ± 0.49	0.15 ± 0.37	0.22 ± 0.59	0.191
Embryo transfer day, *n* (%)							
	3rd day	326 (55.3)	213 (53.3)	90 (51.7)	84 (54.1)	151 (51.0)	0.773
	5th day	263 (44.7)	186 (46.7)	84 (48.3)	71 (45.9)	145 (49.0)	
Clinical pregnancy rates, *n* (%)		194 (32.9)	164 (41.1)	54 (31.0)	56 (36.1)	114 (38.5)	0.048
Live birth rates, *n* (%)		170 (28.9)	151 (37.8)	46 (26.4)	50 (32.2)	101 (34.1)	0.017

E2: estradiol, BMI: body mass index, GnRH: gonadotropin releasing hormone, hCG: human chorionic gonadotropin, P: progesterone.

**Table 2:** Univariate regression analysis for the evaluation of factors influencing live birth rate.

		OR (95% CI)	*p*-Value
Age		0.969 (0.951–0.990)	0.004
Infertility duration		1.002 (1.000–1.005)	0.071
Cycle number		1.008 (0.920–1.108)	0.855
BMI		1.012 (0.990–1.035)	0.311
Basal E2		0.999 (0.993–1.002)	0.668
AFC		1.013 (1.002–1.026)	0.019
Total Gn dose		0.999 (0.999–1.000)	0.055
Duration of stimulation		1.050 (0.984–1.120)	0.187
Endometrial thickness on trigger day		1.020 (0.951–1.083)	0.678
Transferred embryos		1.168 (0.981–1.427)	0.081
Serum E2 on trigger day		1.000 (1.000–1.001)	0.823
Serum P on trigger day		0.975 (0.815–1.169)	0.813
P on date of antagonist initiation		0.988 (0.911–1.158)	0.567
LH on date of antagonist initiation		1.012 (0.893–1.211)	0.735
Serum E2 levels on the day of GnRH ant initiation			
	Group I	0.711 (0.548–0.929)	0.013
	Group II	1	
	Group III	0.637 (0.447–0.948)	0.025
	Group IV	0.821 (0.559–1.197)	0.313
	Group V	0.799 (0.649–1.211)	0.528
Quality of embryos			
	Gr1	1.002 (1.001–1.004)	0.037
	Gr2	1	
	Gr3	0.971 (0.944–0.994)	0.041
Embryo transfer day		0.978 (0.935–1.107)	0.835

E2: estradiol, BMI: body mass index, GnRH: gonadotropin releasing hormone, AFC: antral follicle count, P: progesterone, OR: odds ratio.

**Table 3:** Regression analysis for serum E2 levels after adjusting for age, cycle number, infertility duration, antral follicle count, body mass index, total Gn dosed, quality of embryos, and number of transferred embryos.

	Adjusted *, OR (95% CI)	*p*-Value
Serum E2 Levels on the Day of GnRH Antagonist Initiation		
Group I	0.752 (0.580–0.999)	0.048
Group II	1	
Group III	0.595 (0.388–0.911)	0.011
Group IV	0.728 (0.490–1.112)	0.371
Group V	0.809 (0.590–1.179)	0.429

E2: estradiol, OR: odds ratio; * Adjusted for age, cycle number, infertility duration, antral follicle count, body mass index, total Gn dosed, number of transferred embryos, and quality of embryos.

## Data Availability

All the data underlying this article are available in the text, tables, and references (CrossRef). If further break down is requested, such data will be shared upon reasonable request to the corresponding author.

## Abbreviations

AFC	Antral follicle count
ART	Assisted reproductive technology
BMI	Body mass index
CP	Clinical pregnancy
E2	Estradiol
FSH	Follicle stimulating hormone
GnRH	Gonadotropin releasing hormone
hCG	Human chorionic gonadotropin
IVF	In vitro fertilization
IVF-ET	In vitro fertilization–embryo transfer
LH	Luteinizing hormone
